# The future of data-driven relationship innovation in the microfinance industry

**DOI:** 10.1007/s10479-022-04943-6

**Published:** 2022-09-20

**Authors:** Umme Hani, Ananda Wickramasinghe, Uraiporn Kattiyapornpong, Shahriar Sajib

**Affiliations:** 1 School of Business, Faculty of Business and Law, University of Wollongong, Wollongong, NSW 2522 Australia; 2 UTS Business School, University of Technology, 15 Broadway, Ultimo, NSW 2007 Australia

**Keywords:** Relationship innovation, Big data analytics, Data-driven innovation, Trust, Commitment, Sustained competitive advantage

## Abstract

Data-driven innovation (DDI) initiatives by microfinance institutes have transformed the global poverty alleviation landscape. Despite the fact that relationship building is one of the primary goals of DDI initiatives in microfinance operations, there has been little research on the dimensions of relationship quality. This study examines how DDI initiatives recognize and incorporate relational dimensions in their service offerings to alleviate poverty. Drawing on a systematic literature review, thematic analysis and interviews with 20 microfinance managers, this research explores the relationship quality parameters that need to be leveraged. Grounded in the resource-based theory, the findings of this study confirm trust and commitment as two key relationship capabilities. The findings contribute to a better understanding of how microfinance institutes can use DDI to achieve sustainable competitive advantage.

## Introduction

“Big data can be a powerful tool to help combat poverty. While data helps understand the needs of the poor and design solutions, the technology makes it possible to reach the poor rapidly”, as stated by Grameen Foundation CEO Steve Hollingworth (Wharton, 2019)*.* Financial institutions have had limited success in meeting the requirements of economically active low-income households and micro-enterprises in emerging nations (Fang & Zhang, 2016). The microfinance sector has obtained a license as a formal financial institution that provides credit to low-income borrowers. According to the International Monetary Fund (IMF) report, two hundred million people were not eligible for formal credit before microfinance began (Wharton, 2019). Despite the enormous contribution of microfinance in alleviating poverty and financial inclusion, it constantly faces challenges in maintaining a long-term relationship with its customers (Hani et al., 2021a, 2021b). Microfinance relies on a continuing relationship with its customers for monitoring and collecting repayment (Hani et al., 2021a, 2021b). Relationship innovation can play a crucial role in mitigating this relationship problem, embedding trust, commitment, value, and satisfaction in their offerings (El-Kassar & Singh, 2019; Sänger et al., 2014). We define relationship innovation as a new relationship offering using big data related to microfinance activities (Akhtar et al., 2019).

Big data transforms the global innovation landscape by introducing unique services or processes (Davenport & Kudyba, 2016). Obtaining enormous volumes of client data has grown more accessible, allowing financial services to innovate and differentiate themselves from the competition. Financial services that move from traditional customer analytics to big data analytics integrate a wide range of relationship-oriented data that will benefit from this expansion (Kitchens et al., 2018). Utilizing relationship-oriented big data and analytics to understand the customer better can create more sustainable relationships (Palmatier & Martin, 2019) and result in immediate benefits such as reducing operational costs and risk (Huber & Funaro, 2018). The integration and utilization of such a wealth of relationship-oriented big data have enormous potential for innovation (Kitchens et al., 2018). Data-driven relationship innovation occurs when a service leverages cutting-edge technologies such as artificial intelligence, blockchain, and cloud computing to create innovative relationship offerings through collecting and mining relationship-oriented data, its integration and processing, and analysis and modeling (Akter et al., 2020). Relationship innovation is significant for a sustainable competitive advantage for competing in the current environment and foreseeable future (Kitchens et al., 2018). A relationship-based sustainable competitive advantage is built on the solid foundation of commitment, trust, and reciprocal bonds, which is difficult to copy (Palmatier & Steinhoff, 2019). The transformative applications of big-data analytics leverage those capabilities to innovate relationship-building offerings to build sustainable competitive advantage on their current operations and innovative products, procedures, and business models (Ransbotham & Kiron, 2017).

Big Data is uniquely positioned to minimize the relationship issues inherent in microfinance. It aids in meeting the ever-changing needs and expectations of microfinance customers (Mahajan, 2019). Due to the fact that microfinance is not a new idea, banks already have data to profile and generate archetypes of previously served borrowers (Choo, 2016). This data set contains a wealth of demographic information, such as age, gender, occupation, estimated income, repayment patterns, and meeting center attendance (Nambiar, 2019). Predictive analytics can be an example of big data which can help resolve relational challenges by identifying behavioral trends and patterns. It allows them to profile and generate archetypes of previous borrowers. In addition to anticipating client requirements, these models enable banks to tailor loan programs to specific demographic groups or even individual borrowers(Choo, 2016). Also, several studies, such as Brown et al. (2011), argue that digital platforms provide the most compelling opportunities to connect with customer relationship development and create sustained value. Nambiar (2019) suggests that big data can play an innovative role in reducing operational costs and building relationships while meeting customers' growing needs and expectations in microfinance. In a similar spirit, Bull (2020) states that microfinance will lag further behind more agile competitors the longer it takes to develop these capabilities. Although DDI leveraging relationship quality parameters gains attention to build a sustainable competitive advantage (Akhtar et al., 2019; Neutzling et al., 2018), virtually no research aims to fill this research gap. Thus, this research addresses the following research question:

**How does big data analytics create relationship innovations and sustain competitive advantages in the context of the microfinance industry?**

The current study investigates the links between relationship innovation and big data analytics in the context of microfinance. Drawing on a systematic literature review, thematic analysis, the resource-based theory and interviews with 20 microfinance managers in Bangladesh, this research aims to explore the relationship quality parameters that need to be embedded in service offerings. This research extends resource-based theory by introducing trust and commitment in designing new data-driven services for customers.

## Literature review

### Big data and analytics

Big data is characterized as a voluminous, complex, and diversified data set that traditional information processing software cannot process (Akter & Wamba, 2016). Additionally, big data is characterized by 5Vs: volume, velocity, variety, veracity, and value (Dijck, 2012; Schroeck et al., 2012; White, 2012). The volume refers to the massive amount of data acquired from a variety of data points, the variety refers to the structured and unstructured nature of the data being collected and processed, and the velocity refers to the rate at which the data is collected and managed and analyzed (Dijcks, 2012; Schroeck et al., 2012). Veracity refers to the reliability and integrity of the data sources and the data that has been gathered (White, 2012); lastly, the value implies the informational, strategic, and transactional benefit that can be harnessed through big data (Wamba et al., 2015). In the present digital economy, big data can be a critical resource to achieve a competitive advantage. Therefore, an increasing number of businesses are investing in big data infrastructure in order to identify and capitalize on new business prospects facilitated by data analytics (Schroeck et al., 2012).

Big data analytics (BDA) refers to the advanced analytic tools and methods that can be applied to the diverse and extensive data set to unravel underlying associations, trends, and valuable insights for the business organization. It reduces cost, increases revenue, identifies new opportunities, and offers a superior experience to customers (Davenport, 2014). BDA can be considered as a population of tools that can extract, interpret information and predict decision outcomes (Bose, 2009). Furthermore, analytics deployment may reveal meaningful patterns that can address critical business problems (Demirkan & Spohrer, 2015). Large corporations are adopting "advanced analytics" or "discovery analytics," utilizing a torrent of data for improved marketing perception and planning (Akter & Wamba, 2016; Russom, 2011). Recently, the deployment of AI-enabled analytics has allowed firms to precisely forecast customers' purchase patterns, predict credit fraud, and target individual customers in real-time for marketing purposes (Davenport, 2014). To gain competitive advantages, managers can leverage big data to facilitate their decision regarding the development of new products or services, and the utilization of big data can generate a competitive advantage in the present competitive landscape (Kamioka & Tapanainen, 2014). Therefore, combining big data with analytics tools to result in successful innovation and competitive advantage is a crucial business priority in the current business environment.

### Data-driven innovation

Big data coupled with analytics has paved the way to advance data-driven innovation. Davenport and Kudyba (2016) define DDI as a systematic approach to capturing and creating value through processing the data accessible to the organization. Davenport and Kudyba (2016) articulate DDI as the process of untangling the potential value of data fostering further innovation accompanying the business model, operational, functional, and strategic aspects of an organization. Stone and Wang (2014) suggest that data utilization to facilitate the innovation process to create value is considered DDI. It delivers innovative applications that carry strategic advantages generated from data analytics to enable specific decision-making processes and organizational performance (Akter et al., 2020).Global tech giants have been taking advantage of DDI due to the rapid development in information and communication technologies. They are increasing investment in initiatives driven by big data and AI, robust analytics capabilities, data governance, and building a data culture aligned with organizational capabilities.

Leading technological companies have leveraged data to build a competitive advantage by differentiating their service offering to offer customers a superior experience. For example, Google's target advertising (Hienz, 2014) and LinkedIn's service, such as ‘people you may know,’ can be an example of DDI (Davenport & Kudyba, 2016). Scholars in the field of data-driven innovation have informed in the dominant area such as supply chain management (Sanders 2014), e-commerce (Akter & Wamba, 2016), business intelligence (Chen et al., 2012), and other essential fields. Early research on DDI primarily paid attention to building traditional information products (Browning et al., 2002; Kim et al., 2006; Littler et al., 1995; Meyer & Zack, 1996; Moenaert & Souder, 1990; Nambisan, 2003; Von Hippel, 1998) that did not capture the scope of big data analytics in the DDI processes. In recent times data-driven innovations have effectively been adopted in business activities such as data-intensive product development (Zhan et al., 2019), R&D (Kayyali et al., 2013), and data-driven processes and marketing (Erevelles et al., 2016). The scholarly field of DDI is in an early phase requiring more theoretical and empirical research on data-driven initiatives (Davenport, 2014; Ghasemaghaei & Calic, 2019). Relationship innovation is also a relatively new field of study; therefore, how data-driven innovation can facilitate relationship innovation and create competitive advantage within a microfinance service environment in a developing country context will reveal valuable insights. Table 1 shows the research on data-driven service innovation and the research gaps.

**Table 1:** Important studies on data-driven service innovation

Study focus	Study	Is there any study on relationship innovation in microfinance?	Key findings on data-driven innovation
The potential scope of data analytics in developing data products	Davenport and Kudyba (2016)	No	By combining data analytic capability and increasing valuable data assets, companies can offer value-added information as differentiated products or service offering to generate a higher level of revenue by reaching a more significant customer segment
Personalized recommendation system for an online learning platform	Xiao et al. (2018)	No	Authors suggest that through association rules, collaborative filtering and content filtering, personalized recommendation systems effectively provide the learners with customized assistance to address their individual preferences in an online learning platform
Scope of customer-generated data in digital marketing and service innovation	Balayan and Tomin (2020)	No	The article highlights the current dominant digital marketing practices adopted by platform-based service providers to continuously collect data from users' interactions for monetizing purposes
Application of BDAC on the online streaming platform	Gilmore (2020)	No	The author analyses the underlying mechanism that online-based platforms utilize data produced by the customers' interactions, such as clicks, with a platform such as Netflix to generate customized experiences and leverage the data to refine further and improve algorithms used for the recommendation system
An empirical study on the impact of BDAC on BMI	Ciampi et al. (2021)	No	Big Data Analytics Capabilities (BDAC) offer essential tools and technologies to achieve competitiveness in a highly dynamic marketplace. This study finds a positive impact of BDAC on Business Model Innovation (BMI). It suggests that BDAC has significant potential for creating value for the companies and their stakeholders
Role of BDA in e-commerce	Alrumiah and Hadwan (2020)	No	The authors suggest that electronic vendors utilize Big Data Analytics (BDA) to achieve a competitive advantage and increase revenue outcomes. Further, the study confirms that e-commerce companies aim to understand customers' behavior to improve customer loyalty through processing and analyzing big data
A quantitative study on big data analytic capability on service innovation	Xiao et al. (2018)	No	The authors find a positive impact of Big Data Analytics Capabilities, namely big data analytics personnel capabilities (BDAP) and big data analytics technical capabilities (BDAT), on service innovation mediated by dynamic capabilities. Further, the study highlights the significance of digital platform capabilities in performing service innovation
Consumer trust-building of e-retailers	Chen and Dibb (2010)	No	The authors examined online retailers and found that trust is important in developing favorable behavioral attitudes in customers and intentions towards the e-retailer's website. The study further reveals user-friendliness of the interface, security, assurance of privacy, and superior product information quality as quality features that affect consumers' trust

### Data-driven relationship

The data-driven relationship has emerged as an essential research domain for managing customer relationships in the current business environment. Customers' information from interactions during the service delivery, information regarding the nature of the usage of the service by the customers, or valuable demographic and contextual information on customers' can be leveraged to enhance the relationship quality with the customer (Brown et al., 2011; Gilmore, 2020). In the age of the rapidly changing technological landscape, large organizations have adopted data-driven technologies within their customer relationship management practices to effectively tailor their service according to the customers' requirements (Davenport & Kudyba, 2016). The plethora of customer information has enabled companies to successfully deliver personalized services (Balayan & Tomin, 2020; Xiao et al., 2018). In addition, the emergence of big data analytics to extract actionable insights from big data has enabled present business organizations to perform service innovation more effectively than before (Akter et al., 2020; Zhan et al., 2019). For operational service innovation, by utilizing data generated at various touchpoints during customer interactions, it is essential to adopt an effective digital strategy to deliver customized assistance by recommending a system to enhance the relationship with the customer (Gilmore, 2020; Xiao et al., 2018). A customer-oriented perspective supported by data-driven practices allows financial service providers to identify potential strategies for building a special relationship with the customers to retain them over the long term (Garepasha et al., 2020). Therefore, fostering data-driven relationship innovation will be vital to service organizations' long-term success.

### Data-driven relationship innovation

Innovation focusing on the factors associated with superior customer relationships within present digital communication channels and social media is critical (Garepasha et al., 2020). By leveraging insights from customer data, service companies can gain a higher degree of loyalty, commitment, and trust, resulting in superior firm performance and competitive advantage (Kitchens et al., 2018; Palmatier & Martin, 2019). Akhtar et al. (2019) define relationship innovation as more relevant to the former transformation because it spans numerous aspects (e.g., satisfaction, trust, data, information, and analytics) and enables collaborating partners to develop innovative relationships. The customer relationship of the modern-day relies heavily on big data analytics to gain visibility into their operations and monitor market trends (Hazen et al., 2014). McAfee and Brynjolfsson (2012) noted that data-driven decision-making significantly enhances relationship innovation. It is also worth noting that the power of big analytics can considerably impact the management of several operational activities (Akhtar et al., 2019). For instance, financial institutions can analyze customer requirements, behavior, and sales patterns to aid in precise forecasting and inventory management, supporting innovative relationship development (Akhtar et al., 2019). To appropriately identify the customers' requirements considering the unique contextual factors, it is important to pay serious attention to different relationship quality parameters with the customers through leveraging data-driven tools and technologies.

### Data-driven relationship in financial services

Big data are transforming financial services across the globe. Data analytics and computational power advancements enable businesses to utilize data more efficiently, quickly, reliably, and on a bigger scale (Abraham et al., 2019). To present, research in the financial sector reveals that customer relationship is one of the critical sectors for innovation using big data (Arthur & Owen, 2019; Coumaros et al., 2014; Kitchens et al., 2018; Palmatier & Steinhoff, 2019). Data analytics is being utilized to enhance the client experience facilitated by customized product and service offers. This data analytics includes profiling and segmenting customers, trend analysis, and predictive modeling (Arthur & Owen, 2019; Berry & Linoff, 2004). These initiatives also include identifying potential customers and analyzing marketing campaign success or failure (Rygielski et al., 2002). In the context of B2C relationship quality in online banking services, the authors find that satisfaction, commitment and trust are three dimensions of online relationship quality between consumers and banks (Brun et al., 2014). These dimensions of relationship quality will assist financial institutions in fostering long-term relationships through determining the relational positioning to improve targeted marketing strategies and activities. Within the context of financial services, through investigating the B2C relationship quality of the Islamic banking industry in Malaysia, Haron et al. (2020) find empirical evidence of the partial mediation role of trust between customer satisfaction and loyalty. Further, Amin (2016) investigates online banking services and finds a significant relationship between customer satisfaction, loyalty, and service quality based on an empirical study on online banking customers in a developing country. In a similar notion, Garepasha et al. (2020) investigated B2C relationship quality in online banking services in Iran and found that the customer relationship lifecycle affects customer loyalty on customer relationship quality in online banking services. The study finds that relationship quality becomes a less effective predictor of customer loyalty over time and advises managers to explore alternative marketing actions to retain customers in the long term. Finally, based on an empirical study on trust-building strategies by financial companies, Yousafzai et al. (2005) recommend that situational normality mechanisms and structural assurance impact customers' trustworthiness perceptions that financial institutions can use to develop a portfolio of strategies to build trust with customers. Therefore, adopting a practical digital approach is critical for financial institutions to build superior customer relationships (Sia et al., 2016). Although the above studies have demonstrated the importance of relationship dimensions in online banking using big data, they fail to investigate these relational dimensions through the lens of the microfinance context, which is widely ignored and neglected.

### Data-driven relationship in microfinance

The formation of direct and mutually beneficial relationships has been cited as the essential innovation in microfinance (Baledh & Peña, 2016). The relationship dimension trust has been identified as one of the relationship innovations that aided in reducing information asymmetries and the establishment of compatible incentives, enabling MFls to succeed in providing financial services, particularly lending, to low-income segments of the population (Baledh & Peña, 2016). Brown et al. (2011) suggest that trust and relationships play a vital role in accessing capital for entrepreneurs within complex relationship dynamics of actors in the financial capital industry. Aggarwal (2015) investigates the factors that influence customer trustworthiness in the microfinance industry in developing countries and discovers that lending institutions use borrower demographic information, such as gender, to assess repayment reliability in societies where behavior trust formation processes are prevalent. For example, within the context of social banking, the loan receiver may experience an unexpected circumstance that may affect their capacity to repay the premium (Aggarwal, 2015). Mehta et al. (2011) investigate the significance of big data in increasing commitment and trust among women entrepreneurs in microfinance. The study describes how microfinance institutions create psychological commitment to achieve social and economic objectives through regular communication with the use of cell phones.

The research also finds trust and commitment as two dimensions of microfinance relationships (Cornée & Szafarz, 2014). These dimensions of relationship quality will assist microfinance in mitigating informational opacity and the risk of credit default. However, regular communication with microfinance customer who mostly lives in remote village is often challenging (Choo, 2016). Big data enables microfinance to access customer information, credit score, transaction pattern, and customer behavior pattern (Choo, 2016) and assists in developing trust and commitment building strategies to mitigate credit default risk (Mehta et al., 2011). The empirical investigation by Hani et al., (2021a, 2021b) describes the importance of relationship quality dimensions in recovering the quality of life of the poor customer of microfinance using different platforms such as mobile and face-to-face platforms (Hani et al., 2021a, 2021b). Big data analytics can process such data from various platforms by harvesting information to build relationship innovation (Akhtar et al., 2019), which can help microfinance achieve better visibility, veracity, versatility, and velocity. In addition, microfinance institutions can effectively control risk by tailoring their services to the needs of potential clients (Choo, 2016). Besides, big data can create value for microfinance customers by building trust and loyalty in their relationships (Finnegan, 2014). Despite the prevalence of references to big data in microfinance and relationships, there is a dearth of microfinance-specific research on relationship innovations based on big data.Table 2 shows a literature review of studies highlighting the importance of big data and relationship quality in financial and microfinance services.

**Table 2:** A literature review on big data and relationship quality in financial and microfinance services

Study focus	Study	Context	Key findings and research gaps
Relationship quality dynamics in social banking	Hani et al., (2021a, 2021b)	Social banking	The study identifies respect, reciprocity, and trust as critical relationship quality dimensions. In addition, it discusses how different channels (such as mobile and face-to-face) can be used to continue a long-term relationship. However, it did not discuss relationship innovation using big data
Relationship quality of online banking customers in Spain and Mexico	Olavarría-Jaraba et al. (2018)	Online banking	Considering the context of online channels within an empirical context of the Spanish and Mexican banking industry, the authors find that market orientation, knowledge management, and customer perception about investment in customer relationships positively affect relationship quality. Therefore the findings are essential for long-term relationship management with the customers
Opportunities, dangers, and challenges of using digital technologies and big data in the MFI	Baledh and Peña (2016)	Microfinance	The study discusses the potential to increase efficiency, productivity, and customer service by offering affordable, convenient, and secure MFI services using the widespread use of ICT, such as mobile phones and tablets. However, it does not discuss the innovation of relationships using such technologies
The digital strategy of large financial institution	Sia et al. (2016)	Financial service	Highlighting the customers’ increasing demand for digital products by explicating the case of a large Asian bank DBS, the authors articulate the critical capabilities needed to pursue an effective business strategy for the digital technological landscape
Factors of customer trustworthiness within the Microfinance industry	Aggarwal (2015)	Microfinance	Although the authors confirm the utilization of demographic information of borrowers to determine the trustworthiness of repayment of microcredit loans, the study is not focused on relationship innovation using big data
B2C relationship quality in online banking services	Brun et al. (2014)	Online banking	Based on an investigation of online banking services, the authors find that trust, satisfaction, and commitment are three dimensions of online relationship quality between consumers and banks. These dimensions of relationship quality will assist financial institutions in fostering long-term relationships through determining the relational positioning to improve targeted marketing strategies and activities
Importance of cell phones and relationships for agricultural entrepreneurship in East Africa	Mehta et al. (2011)	Microfinance	Although the study highlights trust as an important dimension of relationship quality and the importance of digital technologies for entrepreneurs in a developing country did not focus on relationship innovation using big data
Relationship marketing within online banking service	Lang and Colgate (2003)	Online banking	The authors highlight the importance of relationship marketing in the financial service industry. The study finds evidence of information technology and information technology channels in fostering solid relationships with customers

## Theoretical underpinnings

The resource-based view (RBV) of the firm emphasizes the possession and utilization of valuable, rare, inimitable, and novel resources for building sustainable competitive advantage (Penrose, 1959; Barney, 1991). In the rapidly changing business environment, it is challenging for companies to sustain a competitive advantage. As a result, it has become critical to focus on intangible assets such as relationships with consumers built on goodwill, trust, dedication, and loyalty (Brown et al., 2011; Hollebeek, 2019). The RBV advocates a foreseeable linkage between resource and organizational value creation leading to superior performance outcomes. Furthermore, a superior relationship with the customers can be leveraged to create value to achieve a competitive advantage that highlights the importance of adopting the RBV for studying relationship innovation within microfinance services.

Relationship with customers is a valuable organizational resource that can potentially contribute to superior organizational performance in various ways. The superior relationship fostered by positive emotions by the front-line employees during interactions with the customers significantly increases customers' trust, loyalty, and excellent organizational performance. Based on empirical investigation on B2C relationship quality, Hollebeek (2019) suggests that engagement between business and customers is a critical factor in fostering co-creation and productive relationships through examining the social media adoption and engagement by business enterprises. The author finds the importance of RBV in studying customer engagement through conceptualizing business-to-customer relationships as a critical resource for enhancing performance. On the other hand, employees' skills and capacity to harness and nurture superior customer relationships through fostering customer engagement are precious in the current business environment (Ranjan et al., 2020).

At present, digital economy data is considered one of the most valuable strategic assets (Morabito, 2015). Balayan and Tomin (2020) highlight the dominant digital marketing practices adopted by platform-based service providers to collect data from users' interactions for monetizing purposes continuously. Further, the rich insights obtained through customer data analysis are critical resources for superior operational and strategic performance outcomes, therefore considered valuable resources by scholars (Alrumiah & Hadwan, 2021). Thus, DDI focusing on relationship innovation will enable organizational members to create valuable intangible resources that can effectively leverage to gain superior financial performance. This study aims at integrating the RBV with the present data-driven relationship innovation research stream to allow superior theoretical underpinning. Shahbaz et al. (2020) emphasize considering BDA as an organizational resource that develops dynamic organizational capabilities such as customer relationship management (CRM). The study reveals that BDA plays a significant mediating function between CRM and a company's sales performance. Finally, following the RBV, Shibin et al. (2020) say that a company's ability to manage its marketing information system positively affects its competitive performance. This effect is partly mediated by marketing analytics and service innovation in a data-rich environment.

## Research approach

Although big data analytics gains momentum in customer relationship literature, it often fails in real-life situations to capture the complexity of an individual's actual behavior (Akter et al., 2020). This investigation adopted a systematic review of the literature with triangulation and thematic investigation of 20 in-depth interviews to produce the dimensions of relationship innovation in microfinance (Akter et al., 2019). The study synthesizes qualitative interviews of practitioners and managers to extract their perspectives on relationship innovation, context, settings, and meanings (Skinner et al., 2000). Thus, in-depth interviews helped triangulate the findings of the systematic literture review (Carter et al., 2014). Furthermore, adopting a scientific approach has offered transparency of the protocol to explicate the scope, criteria, and methodology at every stage of the literature review to ensure accuracy and objectivity (Hossain et al., 2019).

### Literature review strategies

The review procedure begins with the research inquiry: How does big data create relationship innovations in microfinance? This research inquiry facilitated the review procedures to identify the study domain accurately, sources of information, relevant scholarly works, and the criteria determining the omission and addition for the evaluation. The review depicted the insightful and meaningful aspects supported by scientific evidence to offer a practical answer to the research inquiry. This research defined a list of requirements for including relevant articles. Firstly, the time period was 2001 to 2021; hence the search includes publications from 2001 in order to achieve rigor in the big data and microfinance literature. Secondly, the study considers all scholarly articles related to customer relationship quality in financial services as there has been little investigation in this particular empirical domain. Lastly, because the research’s objective is to explore relationship innovation in microfinance through big data, all scholarly articles on big data in microfinance, data-driven service innovation, and customer relationship quality in microfinance were reviewed. A database including the keywords used for searching was: ‘big data’, ‘relationship innovation’ with the words ‘microfinance’, ‘financial service’, ‘banking’, ‘online banking’. Searching for ‘customer relationship in microfinance’ and ‘big data in microfinance relationship’ generated other scholarly articles.

This research began in March 2019 and concluded in November 2021. The electronic databases were exhaustively searched for peer-reviewed academic journal articles and high-quality online content and publications or journals related to microfinance/ financial service relationships from the year 2001 to 2021: ScienceDirect, Business Source Complete, EBSCO Open Access Journals, and ABI/INFORM Complete are highly reputed, widely used and top-ranked journals in social science and business disciplines. Also, other important sources such as; the Web of Science, Scopus, and Google Scholar have also been regarded as the primary source of search. There were 239 peer-reviewed scholarly papers found in the initial search using the keyword, title, and abstract. Only 23 articles were chosen for in-depth examination after cutting down from the original list of 47 articles. The researchers were able to envision a variety of frameworks that pull big data relationship innovation in microfinance as a result of their examination of these studies. The theme analysis section of the literature review is useful for detecting recurring or recurrent connotations of themes across a data set associated with the investigated phenomenon, empirical contexts, or issues.

### Thematic analysis of literature review and in-depth interviews

In accordance with Braun and Clarke (2006), we conducted a thematic analysis of our data. The extensive review of 23 papers yielded a total of two distinct themes. Krippendorff's alpha (or, Kalpha) was estimated in this study to verify the validity of the analysis as a reliable predictor of events. It takes three judges to assess two themes (trust and commitment) and five sub-themes (credibility, customization, caring, affective commitment, and continuance commitment). An inter-rater reliability analysis was conducted using IBM SPSS 26. (Hayes & Krippendorff, 2007). The results report a Kalpha value of 0.82, which is greater than the 0.80 cut-offs, confirming inter-rater reliability (De Swert, 2012).

A qualitative study in the form of in-depth interviews (*n* = 20) was conducted with microfinance managers to validate the findings of the thematic analysis further. The sample’s demographics were diverse in terms of gender, age, education, and profession. The age range of the participants is between 18 and 64 years old, 65% are male, and interviews were conducted via telephone with a duration of approximately 60 min. The sampling was designed following a convenient sampling method, and all the participants were willing to volunteer to participate in this study. We adopted a nonprobability sampling method as it produces rich, insightful, and detailed data to explicate the complexity of the phenomenon and its diversity (Sim et al., 2018). Based on the purposive sampling selection criteria, convenience sampling is critical to the study goal (Sim et al., 2018).

**Fig. 1:**
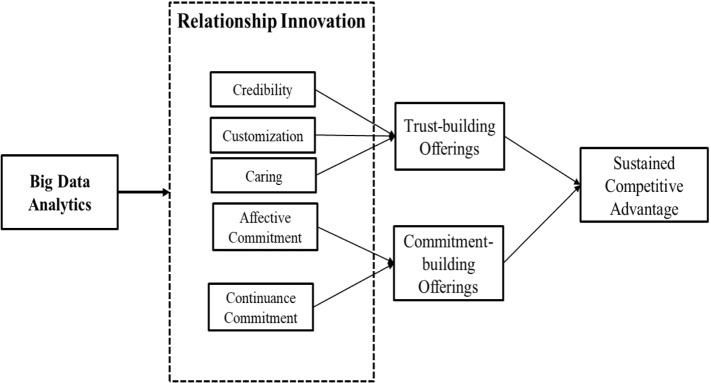
Proposed relationship innovation model using big data analytics

NVivo content analysis software was used in conjunction with manual analysis to capture, transcribe, evaluate and assess the discussions and conversations conducted during the project (Hossain et al., 2019). During the qualitative study, numerous considerations were taken into account. Initially, passages from the early interviews were highlighted. Then, the fundamental aspects of relationship innovation in microfinance, such as trust and commitment, were confirmed. Finally, two independent academic judges reviewed the interview excerpts and uncovered the reoccurring themes within the key dimensions. The findings confirmed the sub-dimensions of trust (i.e., credibility, customization, caring) and commitment (i.e., affective commitment and continuance commitment). NVivo and Microsoft Excel were used again at this stage of the study to estimate inter-rater reliability, which was found to be 0.84, exceeding the 0.80 requirements (Straub et al., 2004). Figure 1 depicts the last two propositions or themes derived from qualitative interviews: trust and commitment, which are congruent with the literature research results. Next, we discuss the proposed relationship innovation model that resulted from the findings of the comprehensive literature review and in-depth interviews.

## Proposed model

We propose a relationship innovation model with two dimensions and five subdimensions based on the results of the previous section. "Trust" is the first dimension, which is subdivided into three subdimensions: credibility, customization, and caring. "Commitment" is the second dimension, which is subdivided into two subdimensions: affective and continuous commitment. The model also proposes that the influence of relationship innovation on microfinance can contribute to sustained competitive advantage (see Figure 1).

### Trust

Trust is one of those resources for an organization that is inimitable, rare, valuable, and non-substitutable (Barney, 1991). Nevertheless, businesses maintain superior performances by utilizing this resource to develop competitive advantages (Srivastava et al., 2001). Specifically, microfinance requires trust to maintain an ongoing relationship where uncertainty or risk is inherent and informational opacity is typical (Hani et al., 2021a, 2021b). Big data can mitigate this informational opacity and uncertainty by providing trustworthy offerings to microfinance customers. We define trustworthy offerings as big data that captures data for delivering credible, customized, and caring offerings for microfinance customers. Our findings align with Hani et al., (2021a, 2021b)’s earlier research. According to the systematic literature review and interviews from the microfinance managers, the study finds that trustworthy offerings consist of credibility, caring and customization.

#### Credibility

Credibility in trust plays a crucial role in extenuating informational opacity and uncertainty associated with microfinance. We define credibility as promises and information provided by microfinance perceived as accurate and can perform the job accurately and effectively (Hani et al., 2021a, 2021b). Customers seek credibility when information or promises become pertinent for actions or decisions yet unknown from personal experience(Wanner & Janiesch, 2019). In microfinance, *the bank’s credibility is indicated by the fact that it is expected not to make any false claim or charge higher interest to their vulnerable customers* (Hani et al., 2021a, 2021b, p. 300). For example, big data analytics can provide up-to-date and real-time data using the bulk amount of text, data, and analysis of customers' responses from online and offline feedback or opinions (Wanner & Janiesch, 2019). Thus, big data can offer various prospects for credible service offerings for microfinance customers, considering these data to mitigate uncertainty and informational opacity among customers*.* The following quote signifies how big data assists in delivering a credible service offering for social banking customers:

*Data can assist us in predicting the number of installments of each customer and correctly analyze credit losses or defaults. Thus, we can effortlessly identify our current and past transactions with our customers, which can help us uphold our integrity among customers and deliver our services efficiently and effectively. (Interviewee # 11).*

#### Customization

Customization is the cornerstone of relationship innovation in which a firm offers tailored services or products to meet the expected needs of heterogeneous customers(Coelho & Henseler, 2012).

Customization in service contributes to trust-building, especially the availability of big data provides organizations new opportunities to customize products or services to ever-demanding customers. Big data enhances an organization's ability to maintain customer relationships by obtaining and analyzing consumer information, permitting more effective firm–customer interactions, and expediting product and service customization (Garrido-Moreno et al., 2014). CRM's data-mining capabilities let businesses better understand their customers' behavior and customize their products and services accordingly (Krasnikov et al., 2009). For example, with the help of big data, ‘BRAC’s microfinance introduced the ‘Client Interaction Point (CIP)’ for readymade garments (RMG) workers in Bangladesh. BRAC realized that although RMG workers are an integral part of the Bangladeshi RMG industry, they are mostly unbanked. They cannot manage time to open a bank account during the working day or compromise a pay cut. Understanding the importance and urgency of this need, BRAC introduced a customized product, “CIP,” a digital financial service (DFS) guaranteeing an end-to-end digital operation process in which customers can directly receive the loan, pay installments and save money through bKsah or cash (BRAC, 2021). In microfinance, we define customization as the bank's ability to tailor products and services to meet the specific demands of each customer (Hani et al., 2021a, 2021b). The importance of big-data analytics in customized service offerings is reflected in the following quote:

*Data allow us to identify trends and patterns concerning current loan information and future loan offerings. In this context, we can use structured and unstructured data in the form of transaction, demographic, psychographic, and socio-economic variables to develop personalized offerings. Managers can use this information to better understand customers' financial circumstances and take necessary actions.(Interviewee #17).*

#### Caring

Caring for microfinance customers is critical because they are vulnerable and encounter various challenging environments (Hani et al., 2021a, 2021b). Customer’s trust in microfinance is embedded in the caring attitude that prioritizes the customers' well-being over the organization’s interests (Ganesan, 1994). It is the degree of willingness to do good for the customers without any profit motive (Mayer et al., 1995). The importance of big data in microfinance for providing caring offerings to vulnerable customers has recently appeared in the current pandemic lockdown in Bangladesh. For example, BRAC microfinance used big data to identify the clients who required emergency money and returned savings during the rapid countrywide lockdown (Gateway, 2020). In the microfinance context, caring is helping the customers in any new circumstances that were not promised before, and there is no intention to make profits.The following quote also apprehends the importance of big data in microfinance for caring offerings:

*We are serving the most disadvantaged populations of society. Unfortunately, natural, political, and environmental instability is often pervasive. Data can allow us to identify the most needed customer and assists us in offering the services to help those customers* ( Interviewee *#* 5)

The qualitative research studies showed that credibility, caring, and customization can be used to predict different types of trust among microfinance customers using big data analytics. Also, the previous qualitative study exhibited that microfinance customers mentioned the importance of customization, caring, and credibility when describing trust (Hani et al., 2021a, 2021b). The notion of trust has been postulated as being the basis of quality interpersonal interactions and a source of competitive advantage for businesses (Tan & Lim, 2009). Trust, after all, gives value to customers and determines their loyalty to a business. Many firms view this client value as critical when looking for innovative strategies to achieve and sustain a competitive edge(Ismail et al., 2017). Thus, the study put forward the following proposition:

##### Proposition 1

 Big data-driven relationship innovation creates trust-building offerings.

### Commitment

One of the most important constructs in relationship innovation is commitment (Akhtar et al., 2019). Commitment, in particular, drives a consumer to loyalty in the service industry. It is characterized as a valuable relationship structure that fosters collaboration (Christine Moorman et al., 1992; Morgan & Hunt, 1994). Porter et al., (1974,p.604) stated, *Organizational commitment is defined in the present context in terms of the strength of an individual's identification with and involvement in a particular organization. Such commitment can generally be characterized by at least three factors: (a) a strong belief in and acceptance of the organization's goals and values; (b) a willingness to exert considerable effort on behalf of the organization; (c) a definite desire to maintain organizational membership.* In line with the definition provided by Porter et al. (1974) and Reichers (1985), we define commitment as the dimension of big data relationship innovation in which microfinance provides commitment building offerings to its customers for establishing organizational value and goals, motivating in accomplishing goals and creating a desire to maintain the relationship. Most successful microfinance operations are primarily attributable to their innovative relationship approach and commitment-building strategy among customers, such as participatory management style, close supervision of the members, and rigorous counseling (Wahid, 1994, p. 36). The effective use of big data analytics promotes customers' commitment to the organization (Garrido-Moreno et al., 2014). It also offers innumerable advantages to the organization by providing ready information about the customer's view at every point of contact (Doherty et al., 2012). Therefore, an organization can identify the crucial commitment-building strategy for microfinance customers. Several studies have considered commitment as a unidimensional construct (MacKenzie et al., 1998; Morgan & Hunt, 1994). Others have captured the multidimensionality of the commitment construct, such as; normative, affective, and continuance commitments (Brown et al., 1995; Gruen et al., 2000; Kumar et al., 1995). Considering our exploratory research and the empirical and theoretical work of Gruen et al. (2000) in relationship marketing, this study proposes a two-component conceptualization of commitment in microfinance relationship innovation—affective commitment and continuance commitment. This study describes commitment as a multidimensional concept where each dimension (affective and continuance) offers various approaches to maintaining a committed relationship (Arcand et al., 2017).

#### Affective commitment

Many studies emphasize affective commitment for a successful long-term engagement with clients (Arcand et al., 2017; Cater & Zabkar, 2009). Affective commitment refers to the positive psychological or emotional bond (Allen & Meyer, 1990; Arcand et al., 2017). It delves into why customers remain in a relationship with the organization. A higher level of participation within the organization offers a higher affective commitment (Gruen et al., 2000). Studies identified that committed customers tend to endorse the organization's well-being by giving something of themselves(Mowday et al., 1982). Thus, we define affective commitment as the degree of the psychological bond of microfinance customers' positive feelings and emotional attachment (Allen & Meyer, 1990; Gruen et al., 2000). Big data can enable microfinance managers to harness unprecedented amounts of customer data and develop knowledge in building such commitment (Garrido-Moreno et al., 2014). The relevance of big data in developing affective commitment and its contribution to business operations is apparent in the following quote:

*Customers' commitment is crucial for the success of microfinance that eradicates poverty. To develop commitment among customers, we allow direct customer participation in our operation process; for example, customers need to form a group of five self-selected members to get a loan. Each group chooses a leader and meeting site, and the necessary installation is collected and shared. This kind of involvement fosters a sense of loyalty and commitment to the group. It makes them feel like a part of it. The stored structured and unstructured data of the customers ( such as the percentage of customer participation, attendance in the weekly meeting, customer problems, and complaints) enables us to identify our committed customers better and reduce operations expenses. (Interviewee # 8).*

#### Continuance commitment

Continuance commitment studies the economic and rational calculation of the losses incurred and benefits sacrificed because of the termination of the relationship (Allen & Meyer, 1990). We define continuance commitment as a relationship innovation in microfinance based on the customer's self-interest and the degree of the psychological bond associated with the cost (social, economic, and status-related) (Gruen et al., 2000) of leaving the institution. The microfinance customers made an initial financial and psychological investment by losing wages, time, transportation, and probable hostility from the community and family (Menon, 2007). It influences the customers to recover their investment (Ferguson & Brown, 1991). In addition, the organization directly controls some benefits and advantages that a customer no longer receives after the termination of the relationship, such as; business-related information, contacts with professionals, recognition for contribution, and professional identifications (Ashforth & Mael, 1989). Big data enables firms to acquire customer behavior and activity knowledge to identify committed customers and develop distinguished operational strategies based on the available knowledge (Garrido-Moreno et al., 2014). The significance of big data in microfinance for building continuance commitment among customers is apparent in the following quote:

*Based on the available data and customer information, we can offer services or products to continue with us. For example, our recorded data tell us which customer diligently paid their installments for the last five years. As a recognition, we offer special loans to our committed customers such as educational loans to the kids, business loans for the partners, and home improvement loans…. (Interviewee # 5).*

The qualitative findings indicated that using big data analytics, affective and continuance commitment can predict various types of commitment among microfinance consumers. Commitment motivates consumers to be loyal. It is a beneficial relationship structure that promotes collaboration (Christine et al., 1992; Morgan & Hunt, 1994). Committed customers are willing to invest in maintaining and developing close relationships (Ismail et al., 2017). Such investment and collaboration may increase efficiency, reduce operational costs, improve financial output, and increase microfinance's competitiveness. Accordingly, the following hypothesis is proposed in this study:

##### Proposition 2

Big data-driven relationship innovation creates commitment-building offerings. Impact of relationship innovation on microfinance’s sustained competitive advantages.

This article argues that the influence of relationship innovation on microfinance can contribute to long-term competitiveness. Innovation such as relationship innovation enables microfinance to identify and develop valuable new offerings such as trust and commitment building offerings that help microfinance establish and maintain a sustainable competitive advantage. It also can direct to new, customized solutions that distinguish microfinance from its competitors. Both theory and practice strongly reflect the underlying innovation process using relationship offerings as a sustainable competitive advantage (Palmatier & Steinhoff, 2019). The resource-based view (RBV) is a theoretical paradigm for investigating how microfinance generates strategic resources using big data that benefit customers (Nyaga & Whipple, 2011). According to the RBV, microfinance can gain a competitive edge when it builds relationships that are distinct from its competitors and difficult to imitate (Barney, 1986). Relationship innovation using big data can be a source of successful competitive advantage if that relationship offering is valuable, rare, and imperfectly imitable. Microfinance can be more competitive in the long run if it makes meaningful, unique, or hard-to-copy relationship offerings like trust and committed service. The qualitative findings of this study have significant implications for current arguments about the ability to create relationships to improve operational and marketing effectiveness in turn (Smircich, 1983; Tichy, 1983). This logic implies that if microfinance can provide such relations to its customers that others can not easily imitate or modify, such relationship offerings can generate sustained competitive advantage. Thus we posit that:

##### Proposition 3

Big data-driven relationship innovation influences overall sustained competitive advantage.

## Discussion

### Theoretical contribution

Several theoretical contributions are made in this paper. In developing the theoretical model, this research considers the resource-based view (RBV) (Barney, 1991; Wernerfelt, 1984) to explain the relationship between institutional data and sustained competitive advantage. This study demonstrates that organizations can harness performance by defining the underlying potential of data obtained through customer interactions and leveraging that data to innovate appropriate relationships with customers within a micro-credit context. Therefore, following the RBV theory, the relational capital of social business, specifically microcredit institutions, utilizes valuable, rare, inimitable, and novel resources for building sustainable competitive advantage (Barney, 1991; Hollebeek, 2019).

RBV is a theoretical framework to identify the critical organizational resources of concern that can be important for the company’s strategic benefit. Within the empirical context, the big data generated through transactional and relational engagement with the customers are proved to be of serious value to delivering service innovation leading to superior customer experience, customer retention, and enhanced relationship quality. Considering the context of the microfinance industry during the Covid-19 pandemic, a financial service provider needs to engage with the customers to anticipate diverse potential causes of financial hardship. Different hardship situations may necessitate recognizing specific emotional and psychological needs fulfilled by positive emotions and skills of front-line employees, such as empathy, compassion, and assurance, to offer assistance during a crisis (Ranjan et al., 2020). Appropriate recognition of these emotional aspects of customer requirements through analyzing available data will play a positive role in retaining customers with a higher degree of trust, commitment, or loyalty; these findings align with the previous empirical studies (Brown et al., 2011; Hollebeek, 2019).

This study demonstrates how managers may effectively deliver service innovation or a superior relational experience to customers through meticulous data analysis, resulting in long-term improved performance outcomes within the context of data-driven relationship innovation. As a result, this study's findings emphasize the need to develop quality relationships for effective customer interaction, consistent with past academic recommendations. (Ranjan et al. 2020). The findings of this study contribute to the growing scholarly domain of big data analytics by demonstrating the linkage between big data analytics and sustainable competitive advantage. Furthermore, this study contributes to the empirical findings that big data is a strategic asset (Morabito, 2015; Alrumiah & Hadwan, 2021; Balayan & Tomin, 2020; Shahbaz et al., 2020). Following the viewpoint of a data-rich environment, this study validates the value of big data in delivering sustainable competitive advantage within a novel empirical context of microcredit financial institutions. The findings of this study have made essential contributions in the scholarly field such as Data-Driven Innovation, Data-Driven Relationship innovation (Akter et al., 2020; Davenport & Kudyba, 2016) through applying a resource-based view in these crucial fields of studies.

### Managerial implications

This study recommends several managerial implications of relationship innovation using big data in microfinance. *First*, managers in microfinance can benefit from data-driven relationship innovation by assessing the psychometric evaluations of relationship quality. Data-driven relationship offerings can evaluate the applicant's responses, allowing information to be captured that might assist in predicting loan repayment behavior, such as the applicant's views, commitment, and trust. *Second*, relationship innovation can aid managers in identifying customer behavior patterns. The integration of customer behavior patterns and crucial business data can be helpful for managers in the creation of application scorecards that can be used for bias-free customer selection. Managers can make credit decisions in a minute to evaluate this customer behavior pattern and reduce the overall operational cost. *Third*, client heterogeneity is the biggest challenge in microfinance. Relationship innovation in big data can play an innovative role in microfinance institutions by fulfilling diverse customer expectations and demands. The microfinance industry will be able to deliver services tailored to its customers' needs based on consumer demands revealed through analytics such as customer segmentation, price modeling, and data modeling. *Fourth*, relationship innovation using big data can estimate portfolio behavior across many geographies, allowing them to analyze defaults and credit losses more precisely. As a result, microfinance institutions can select the portfolio that best suits their financial offerings and gain sustained competitive advantages. *Finally*, managers can evaluate relationships to determine their future benefits by assessing relationship quality. Data analytics exhibits the relationship quality by identifying the relational dimensions of commitment and trust of a customer. Microfinance should consider that relationship innovation can demonstrate relationship quality and deliver advantages while also recognizing that relationships that fail to create relationship quality will not produce the desired results. Relationship quality can positively impact operational and strategic success, regardless of the type of relationship.

## Conclusion

Microfinance appears to be trapped in a bygone era of service delivery, where little seems to have changed even though technology has fundamentally altered retail financial services. Microfinance needs to embrace big data to sustain itself in the competitive age. To gain a sustained competitive advantage, it must adopt big data analytics to compete in this immense competitive era, from innovative product design to customer relationship management, operational efficiency, and portfolio growth (Bull, 2020; Nambiar, 2019). Technology should not replace relationships but rather facilitate the development of the authentic and profound relationship between the MFI and its clients as it expands its operations. Especially relationship innovation using big data can provide sustained competitive advantages by identifying various relational dimensions. Rather than considering a replacement for face-to-face relationships, big data analytics should be viewed as additional advantage microfinance may use to build a strong relationship with its customers.
